# Probabilistic Computation in Human Perception under Variability in Encoding Precision

**DOI:** 10.1371/journal.pone.0040216

**Published:** 2012-06-29

**Authors:** Shaiyan Keshvari, Ronald van den Berg, Wei Ji Ma

**Affiliations:** Department of Neuroscience, Baylor College of Medicine, Houston, Texas, United States of America; Bielefeld University, Germany

## Abstract

A key function of the brain is to interpret noisy sensory information. To do so optimally, observers must, in many tasks, take into account knowledge of the precision with which stimuli are encoded. In an orientation change detection task, we find that encoding precision does not only depend on an experimentally controlled reliability parameter (shape), but also exhibits additional variability. In spite of variability in precision, human subjects seem to take into account precision near-optimally on a trial-to-trial and item-to-item basis. Our results offer a new conceptualization of the encoding of sensory information and highlight the brain’s remarkable ability to incorporate knowledge of uncertainty during complex perceptual decision-making.

## Introduction

The sensory information used by the brain to infer the state of the world is noisy: when the same stimulus is presented repeatedly, the neural activity it elicits varies considerably from trial to trial [1], [2], [3], [4]. As a consequence, an observer’s measurement of a task-relevant stimulus feature varies as well. The quality of the sensory information can be numerically expressed as precision. For instance, when the measurement follows a Gaussian distribution, precision could be defined as the inverse of the variance of this Gaussian.

Models of perception routinely assume that the precision with which a task-relevant stimulus feature is encoded is constant as long as the stimulus is held constant [5]. It is questionable, however, whether this assumption is justified, considering that factors such as fluctuations in alertness [6], configural effects [7], [8], and covert shifts of attention [9], [10] could make precision variable. If all factors were known and quantifiable, encoding precision could be specified exactly for each stimulus on each trial. However, as long as we are not able to model each possible contributing factor, it may be best to model precision as a random variable [11]. For example, the inverse variance of a Gaussian noise distribution could be drawn from a gamma distribution.

If encoding precision is a random variable, then the measurement of a task-relevant stimulus feature follows a doubly stochastic process. This idea translates to the level of neural coding, where a population pattern of activity could be Poisson-like with a mean amplitude (gain) that itself follows some other distribution. Recent physiological studies have reported evidence for doubly stochastic processes in cortex [12], [13], [14], [15].

In the optimal-observer models of many tasks, precision does not only appear as part of the encoding model (a description of how measurements are generated), but also in the observer’s decision rule (a description of how measurements are transformed into a decision). In other words, in some tasks, in order to be optimal, an observer must take into account precision even if precision varies unpredictably across stimuli and trials. To distinguish this type of computation from computation in which the observer can be optimal using only a point estimate of each stimulus feature, we use the term “probabilistic computation” [16]. At the neural level, probabilistic computation suggests that populations of neurons encode and compute with probability distributions over stimulus features [16], [17], [18], instead of only point estimates.

Psychophysical evidence for probabilistic computation has been found in cue combination tasks [19], [20], [21] as well as more complex categorization tasks [22], [23]. In these experiments, the encoding precision of the task-relevant feature was manipulated by varying a reliability parameter, for example the size of a blurred disc if its location is task-relevant, or contrast of a bar if its orientation is task-relevant. Since we propose here that factors other than this reliability parameter also contribute to variability in precision, the question arises whether observers optimally take into account this additional variability.

Here we use a visual change detection task [24], [25], [26] to study whether precision is variable for a given value of the reliability parameter and whether observers take any variability in precision (whether or not due to the reliability parameter) into account optimally. Observers reported whether a change in the orientation of a stimulus occurred between two displays that each contained four stimuli (items). The reliability of the orientation information was controlled by shape and was randomly chosen for each stimulus. We pitted an optimal-observer model in which precision is completely determined by shape (“equal precision”) against one in which there is additional variability (“variable precision”). Both models assume that precision is known and optimally taken into account by the observer on an item-by-item and trial-by-trial basis. We compare these two models to several suboptimal models, where suboptimality can be caused by two factors. First, the observer might make a wrong assumption about precision. For example, if precision varies across stimuli at different locations, the observer might assume a single value of precision for all stimuli instead of using the individual values. Second, the observer might use a suboptimal decision rule instead of the optimal rule to integrate information from different locations. Considering all combinations of model elements – equal or variable precision, various observer assumptions about precision, and two possible integration rules – we arrive at a total of 14 models. We find that the empirical data for each individual subject are best described by the model in which precision is variable, the observer knows precision on an item-by-item and trial-by-trial basis, and uses the optimal integration rule.

## Results

### Experiment

**Figure 1:**
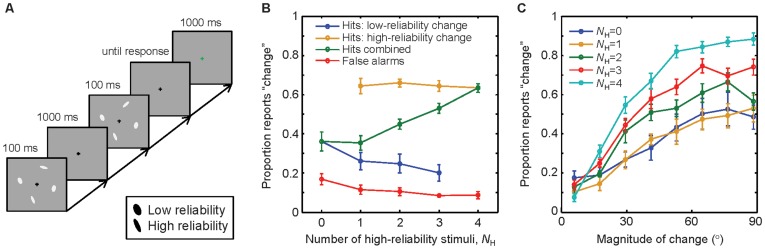
Change detection under varying reliability. A, Schematic of the trial procedure. Stimulus reliability was controlled by ellipse elongation. Set size was always 4. B, Hit and false-alarm rates as a function of the number of high-reliability stimuli (long ellipses), *N*_H_. Hit rates are split out by whether the changing ellipse had high or low reliability. The Z-shape formed by the yellow, green, and blue lines is an instance of Simpson’s paradox (see Results). C, Proportion of “change” reports in change trials as a function of the magnitude of change, for different values of *N*_H_. Error bars represent ±1 s.e.m.

Subjects were presented with two consecutive displays, each presented for 100 ms and separated by a 1-second blank screen. Each display contained a set of four randomly oriented ellipses that were identical between both displays except that with 50% probability, exactly one ellipse changed orientation between the first and the second screen (Fig. 1A). The magnitude of a change, if present, was drawn from a uniform distribution. On each trial, we first randomly chose the number of high-reliability stimuli (0 to 4, with equal probability); then, we randomly chose which of the stimuli had high reliability. Reliability was controlled by shape: high-reliability ellipses were more elongated than low-reliability ones, but had the same area. Subjects indicated whether or not a change occurred.

As expected, subjects became better at detecting a change as the number of high-reliability stimuli, denoted *N*_H_, increased (Fig. 1B). While we did not find a significant effect of *N*_H_ on the false-alarm rate (one-way repeated-measures ANOVA, *F*(2.3,18.6) = 2.9, *p* = 0.08; degrees of freedom were corrected using Greenhouse-Geisser estimates of sphericity), the effect of *N*_H_ on the hit rate was significant (*F*(1.7,13.9) = 25.1, *p*<0.001). This shows that our reliability manipulation was effective. Mean accuracy exceeded chance at every value of *N*_H_ (*t*(8)>5.5, *p*<10^−3^).

When we separate hit trials by the reliability of the changing stimulus, we see a distinctive Z-shaped pattern (Fig. 1B). The hit rate conditioned on the change being in a low-reliability stimulus decreases monotonically with *N*_H_ (*F*(3,24) = 9.7, *p*<0.001). We did not find an effect of *N*_H_ on the hit rate conditioned on the change being in a high-reliability stimulus (*F*(1.4,11.6) = 0.20, *p* = 0.75). It might be counterintuitive that the low-reliability hit rate decreases and the high-reliability hit rate is flat, yet the unconditioned hit rate increases. This effect is an instance of Simpson’s paradox [27]. The apparent contradiction is resolved by realizing that the relative contributions of the conditional rates change with *N*_H_: the higher *N*_H_, the larger the proportion of trials that fall in the high-reliability-change category. The Z-shaped pattern in our data confirms a prediction from an optimal model of a change discrimination task [28] (elaborated below).

Next, we binned change trials by magnitude of change (8 bins) (Fig. 1C). A two-way repeated-measures ANOVA reveals significant main effects of magnitude of change (*F*(7,56) = 109.0, *p*<0.001) and of *N*_H_ (*F*(1.9,15.2) = 24.4, *p*<0.001) on the proportion of “change” reports, and a significant interaction (*F*(28,224) = 5.4, *p*<0.001). This indicates that larger changes are easier to detect.

### Models

**Figure 2:**
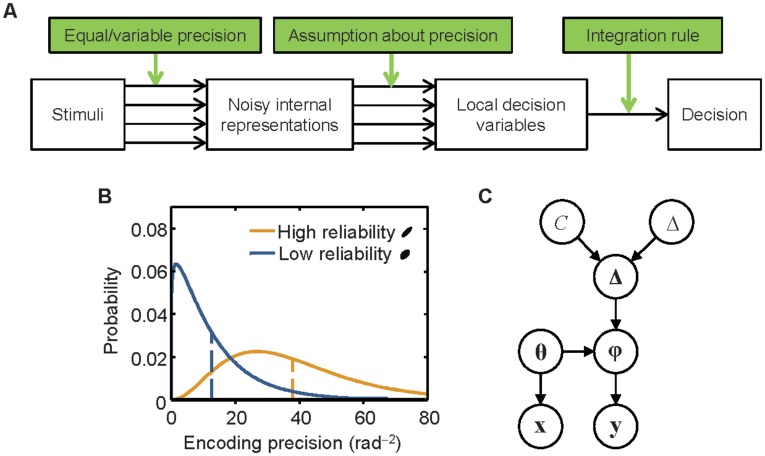
A, Flow diagram of the decision process. Models differ along three dimensions: whether precision is equal or variable, the observer’s assumption about precision, and the observer’s integration rule. B, Examples of probability density functions over encoding precision for a high-reliability and a low-reliability stimulus (long and short ellipse, respectively) in the variable-precision model. Dashed lines indicate the means. C, The generative model shows statistical dependencies between variables. *C*: change occurrence (0 or 1); Δ: magnitude of change; **Δ**: vector of change magnitudes at all locations; **θ** and **ϕ**: vectors of stimulus orientations in the first and second displays, respectively; **x** and **y**: vectors of measurements of the stimulus orientations. The spatial, temporal, and structural complexities of the task can be recognized in the vector nature of the orientation variables, the two “branches”, and the number of layers, respectively.



We model the observer’s decision process as consisting of an encoding stage and a decision stage (Fig. 2A). In the encoding stage, precision is either completely determined by stimulus reliability (“equal precision” or EP), or a random variable itself (“variable precision” or VP). Precision is technically defined as Fisher information (see Methods) and denoted *J*. For a given value of precision, *J*, the measurement *x* of an orientation *θ* follows a probability distribution *p*(*x*|*θ*;*J*). For this distribution, we assume a circular Gaussian (Von Mises) distribution, characterized by a concentration parameter *κ* that corresponds one-to-one with precision (see Text S1 and Fig. S1). When precision is variable (VP), the measurement of a stimulus over many trials is described by a doubly stochastic process, formalized as the following integral:

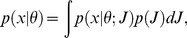
(1)where *p*(*x*|*θ*;*J*) is again the Von Mises distribution and the variability in *J* itself, *p*(*J*), is modeled as a gamma distribution (Fig. 2B). The distribution in Eq. (1) is a mixture of an infinite number of Von Mises distributions, each with its own precision; it is a circular analog of the Student t-distribution.

In the decision stage, the Bayes-optimal observer computes on each trial the probability that a change occurred and responds “change” if this probability is greater than 0.5. This is equivalent to responding “change” when.
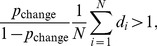
(2)


where *p*_change_ is the observer’s prior belief that a change occurred, *N* is the number of stimuli, and *d_i_* is the local decision variable (i.e., the posterior probability ratio of change occurrence at the *i* ^th^ location, denoted *d_i_*; see Text S1 for derivation).
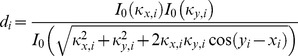
(3)


where *x_i_* and *y_i_* are the measurements of the *i* ^th^ stimulus in the first and second displays, respectively, *κ_x,i_* and *κ_y,i_* are the corresponding concentration parameters of the noise, and *I*_0_ is the modified Bessel function of the first kind of order 0. Eq. (3) represents “weighting” by encoding precision (through *κ_x,i_* and *κ_y,i_*) on a trial-by-trial and item-by-item basis, in a way analogous to but more complex than cue combination. It is crucial that the optimal observer knows precision, *J*, and therefore *κ*, for each display and each item on each trial. Thus, even though Eq. (1) describes a doubly stochastic process over many trials, the optimal observer on a single trial knows the exact conditioned distribution *p*(*x*|*θ*,*J*).

In the decoding stage, the models we consider differ along two dimensions that can be understood in the context of Eqs. (3) and (2), respectively. The first dimension concerns the assumption that the observer makes about encoding precision:

1. no assumption: complete knowledge of an item’s precision on each trial, i.e. the optimal model;
2. the assumption that precision is completely determined by shape, ignoring any other variability (suboptimal);
3. the assumption that precision is equal to the average precision across the display (which will vary across trials), reflecting a “gist” representation of precision (suboptimal);
4. the assumption that precision is equal throughout the experiment, thus ignoring both variations in shape and other variability (suboptimal).

If encoding precision is equal (EP), assumptions 1 and 2 are equivalent, because there is no additional variability to ignore. Assumptions 2 to 4 are formalized as variants of Eq. (3) in which the trial-to-trial and item-to-item concentration parameters are replaced by values that are solely determined by stimulus reliability, by the average value in the display, or by a single value throughout the experiment, respectively.

The second dimension along which the models differ is the integration rule that the observer applies to the local decision variables, *d_i_*. Specifically, besides the optimal rule, Eq. (2), we consider the suboptimal “Max” rule, according to which the observer responds based on the largest local decision variable. The Max decision rule is 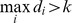, with *k* a constant criterion. The Max rule has been used widely in signal detection theory models of visual search and is considered a reasonable description of human search behavior [29], [30], [31], [32] (but see [22]). The Max model together with the assumption of single precision (Assumption 4) is equivalent to the (also suboptimal) maximum-absolute-differences model we introduced for change detection in earlier work [33] (see Text S1). In total, this produces (4+3)⋅2 = 14 models, listed in Table 1. The number of free parameters ranges from 3 to 5.

**Table 1:** List of models considered.

Model	Precision	Local decision variable (*d_i_*)		Decision rule	#Pars
VVO	variable	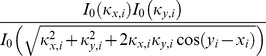	with *κ_i_* the actual value at the *i* ^th^ location	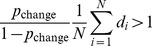	_4_
VEO	variable	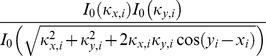	with *κ_i_* either *κ*_low_ or *κ*_high_	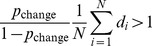	_4_
VAO	variable	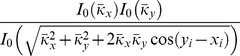	where 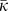 is an average over locations	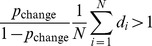	_4_
VSO	variable	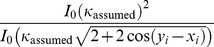		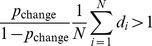	_5_
VVM	variable	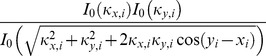	with *κ_i_* the actual value at the *i* ^th^ location	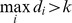	_4_
VEM	variable	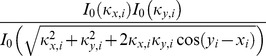	with *κ_i_* either *κ*_low_ or *κ*_high_	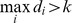	_4_
VAM	variable	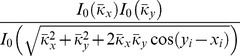	where 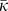 is an average over locations	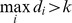	_4_
VSM	variable	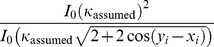		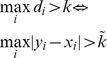	_4_
EEO	equal	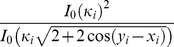	with *κ_i_* either *κ*_low_ or *κ*_high_	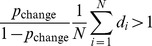	_3_
EAO	equal	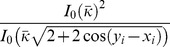	where 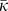 is an average over locations	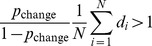	_3_
ESO	equal	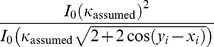		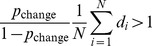	_4_
EEM	equal	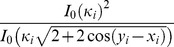	with *κ_i_* either *κ*_low_ or *κ*_high_	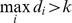	_3_
EAM	equal	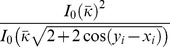	where 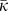 is an average over locations	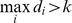	_3_
ESM	equal	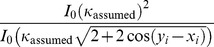		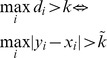	_3_

The first letter stands for variable (V) or equal (E) encoding precision. The second letter indicates the observer’s assumption about encoding precision (V: variable; E: equal; A: sample average over locations; S: single value). The third letter stands for the optimal (O) or Max (M) integration rule. The equivalences (⇔) in the VSM and FSM models are explained in the Text S1; the notation |⋅| denotes circular distance.

### Model Comparison

**Figure 3:**
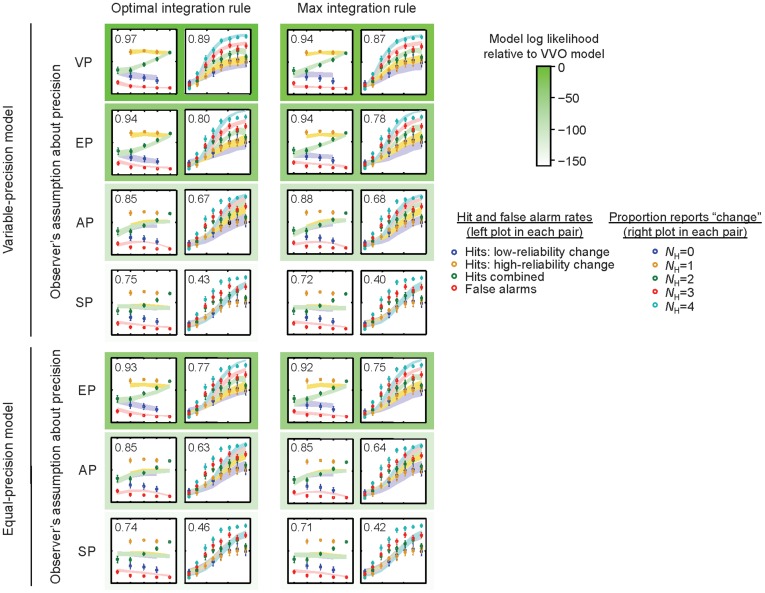
Fits of all 14 models to the data in Fig. 1B-C (axis labels and scales as there). VP  =  variable precision; EP  =  equal precision; AP  =  average precision; SP  =  single precision. Error bars and shaded areas represent ±1 s.e.m. in the data and the model, respectively. The number in each plot is the *R* ^2^ of the fit (for the left plot in each pair, computed over false-alarm rates and unconditioned hit rates). Frame color indicates model goodness of fit relative to the winning model, as obtained from Bayesian model comparison (Fig. 4).

We compared the models in two ways. First, we fitted each model’s parameters using maximum-likelihood estimation and computed *R* ^2^ for the fits to the data in Fig. 1B-C (Fig. 3). The winning model was the one in which encoding precision is variable, observers optimally weight observations by their encoding precision, and they use the optimal rule for integrating information across locations (the VVO model from Table 1). This model had the highest goodness-of-fit for hit and false-alarm rates (*R* ^2^ = 0.97), as well as for psychometric curves (*R* ^2^ = 0.89). Maximum-likelihood estimates of model parameters are given in Table S1.

**Figure 4:**
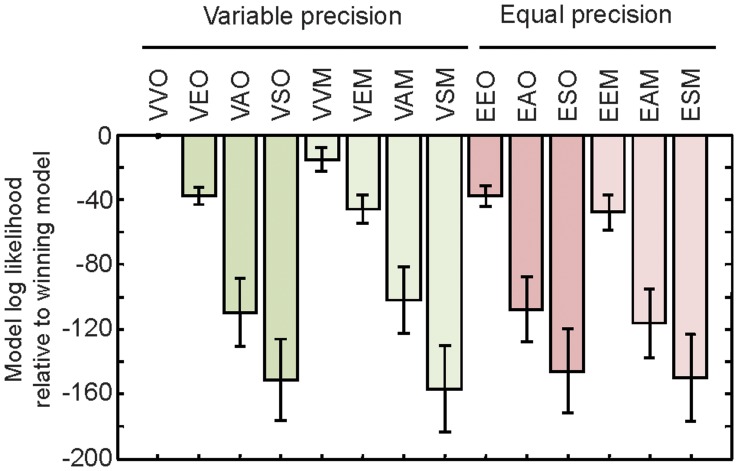
Log likelihood of each model relative to the VVO model. Negative values indicate that the model is less likely than the VVO model. Error bars represent s.e.m. Abbreviations and color scheme are as in Table 1.

Second, to distinguish the models in a more powerful way, we performed Bayesian model comparison [34]. This method computes the average likelihood over all parameter combinations, thereby automatically correcting for the number of free parameters (see Online Methods). The VVO model is the clear winner for each of the 9 subjects individually. Bayesian model comparison revealed that the log likelihood of the VVO model exceeds that of the next best model (VVM, which uses the Max rule) by the decisive difference of 15.4±17.3 (mean and s.e.m.) log likelihood points (Fig. 4).

The VVO model exceeds the EEO model – the best equal-precision model – by 36.3±6.3 log likelihood points, suggesting variability in encoding precision. To confirm that this advantage is not due to unmodeled noise at the decision level (the last two steps in Fig. 2A), we tested two EEO model variants that included such noise. In the first variant (“local decision noise”), we added zero-mean Gaussian noise with standard deviation *σ*_local_ to the log of the local decision variable, *d_i_*. In the second variant (“global decision noise”), we added the same type of noise (with standard deviation *σ*_global_) to the log of the left-hand side of Eq. (2). The best-fitting values were *σ*_local_ = 0.34±0.04 and *σ*_global_ = 0.30±0.08. These values are small given that log decision variables generally ranged from about −4 to 20. Furthermore, we computed the model likelihoods of these two variants, and compared them to that of the winning model, VVO. The EEO models with local and global decision noise had log likelihoods of −37.1±7.0 and −38.2±7.0 relative to VVO, respectively. Moreover, the VVO model described the data better than both noisy models in all nine subjects individually. Thus, decision noise cannot account for the difference between the VVO and EEO models.

### Simpson’s Paradox

As Fig. 3A shows, the VVO model accounts for the characteristic Z-shape in the hit rates. The intuition behind the Z-shape in the context of the VVO model – and in fact any model that weights observations by their encoding precision – is as follows. The unconditioned hit rate increases with the number of high-reliability stimuli, *N*_H_, because more information is available in the measurements, and the observer utilizes this information. The hit rate conditioned on the changing item having low reliability decreases with increasing *N*_H_ because a higher value of *N*_H_ means that more non-changing items have high reliability. Since in the VVO model, more precise measurements influence the decision more strongly, the overall evidence for “no change” becomes stronger and subjects become less likely to report “change”. Our result confirms a prediction from an earlier Bayesian model of change discrimination [28] and provides additional evidence for probabilistic computation by humans in change detection.

## Discussion

We have found that in detecting a change among multiple stimuli: a) the encoding precision of a stimulus is variable even for a given value of stimulus reliability; b) observers near-optimally take into account both variations in stimulus reliability and the additional variability. These results raise several issues.

First, we modeled the distribution of encoding precision as a gamma distribution, with precision being independent across locations and trials. While this choice was convenient and led to good fits, alternatives to the gamma and independence assumptions must be considered.

Second, what causes variability in encoding precision? Several possible factors were mentioned in the introduction. In addition, the precision of memorized items could decay in variable ways, or precision could simply depend on the task-relevant feature value [35]. The relative contributions of these factors remain to be determined.

Third, variability in precision may have implications for encoding models in other tasks. It could potentially account for subject responses that are usually modeled as lapses, since those correspond to a precision of zero. Moreover, in cue combination, it has been suggested that sensory noise is best described by a mixture of a Gaussian and a uniform distribution [36] or of two Gaussian distributions [37]. These mixture models can be regarded as approximations to a full-fledged doubly stochastic process as in Eq. (1), since the mixture components correspond to two different values of precision.

Fourth, how variability in precision can be recognized in neural activity depends on the neural coding scheme one subscribes to. In the framework of Poisson-like probabilistic population codes, variability in encoding precision might correspond to variability in population gain [18], [38]. There is initial evidence that gain does vary [12], [13], [14], and this variability might in part be due to attentional factors [39], [40], [41], [42]. Neuroimaging studies have found that trial-to-trial fluctuations in perceptual performance correlate with fluctuations in stimulus-independent, ongoing neural activity in dorsal anterior cingulate cortex, dorsolateral prefrontal cortex, and dorsal parietal areas [43], [44]. This activity might in part reflect the attentional state of the observer, in which case their fluctuations might partially account for variability in precision.

Fifth, how can a neural population “know” encoding precision for use in decision-making? Again in probabilistic population coding, a neural population encodes on each trial a full likelihood function over the stimulus, whose inverse width represents the precision/certainty associated with that stimulus on that trial [18]. Thus, encoding precision is implicitly known on a trial-by-trial basis and can be used in downstream computation. A next step would be to use probabilistic population codes to design a neural network that takes Poisson-like representations of the individual stimuli in both displays as input and has an output layer that encodes the probability that a change occurred (potentially in the medial temporal lobe [45]).

Our work illustrates a new role for change detection in psychology. Traditionally, change detection has only been used to probe capacity limitations in short-term memory [25], [46], [47], [48]. Viewing change detection as inference on noisy sensory measurements is relatively new [33]. Here, we have demonstrated the use of change detection in studying whether the brain computes with probability distributions. Behavioral evidence for probabilistic computation had so far been largely limited to tasks with relatively simple statistical structures, such as cue combination. Change detection is a case study of complex inference, because of the presence of multiple relevant stimuli (spatial complexity), because stimulus information must be integrated into an abstract categorical judgment (structural complexity), and because perception interacts with visual short-term memory (temporal complexity).

A final caveat. It is tempting to equate optimality with the notion that the brain computes with probabilities on an individual-trial basis (probabilistic computation). These are, however, orthogonal notions [16], [49]. In some tasks, such as judging whether an oriented stimulus is tilted to the left or to the right, optimality can be attained using only point estimates and does not require trial-by-trial representations of probability. Conversely, an observer might take into account precision – and perhaps represent probability – on a trial-by-trial and item-by-item basis, but do so in a suboptimal way. Here, we have provided evidence for both optimality and probabilistic computation in change detection. To test for probabilistic computation, we varied reliability unpredictably without giving trial-to-trial feedback, and compared models in which the observer does or does not take into account precision on a trial-by-trial and item-by-item basis. To test for optimality, we compared the optimal decision rule against a plausible suboptimal one, the Max rule. Thus, we were to some extent able to disentangle Bayesian optimality from probabilistic computation. We speculate that as task complexity increases, optimality will break down at some point, but probabilistic computation continues to be performed – in other words, humans are suboptimal, probabilistic observers.

## Methods

### Stimuli

Stimuli were displayed on a 21″ LCD monitor at a viewing distance of 60 cm. Each stimulus display contained four oriented ellipses. Two types of ellipses were used: “long” and “short” ones. “Long” ellipses had minor and major axes of 0.37 and 1.02 degrees of visual angle (deg), respectively. “Short” ellipses had the same area, but their elongations were determined separately for each subject (see Procedure). On each trial, ellipse centers were chosen by placing one at a random location on an imaginary circle of radius 7 deg around the screen center, placing the next one 90° counterclockwise from the first along the circle, etc., until all four ellipses had been placed. This spacing was sufficiently large to avoid crowding effects. Each ellipse position was jittered by a random amount between −0.3 and 0.3 deg in *x*- and *y*-directions (independently). Stimulus and background luminances were 95.7 and 33.1 cd/m^2^, respectively.

### Subjects

Nine subjects participated (6 naïve, 3 authors; 1 female). All were between 22 and 32 years old and had normal or corrected-to-normal vision. The study was approved by the Institutional Review Board for Human Subject Research for Baylor College of Medicine; all subjects gave written informed consent.

### Procedure

There were three types of trial blocks: testing blocks, practice blocks, and threshold blocks. In each testing block, a trial began with a blank screen displaying a central fixation cross for 1000 ms. The first stimulus display was presented for 100 ms, followed by a delay period of 1000 ms, followed by a second stimulus display for 100 ms. On each trial, the number of long ellipses was chosen randomly with equal probability from 0 to 4. The locations of the long ellipses were chosen randomly given the constraint of their total number; all other ellipses were short. The orientation of each ellipse was drawn independently from a uniform distribution over all possible orientations. The second stimulus display was identical to the first, except that there was a 50% chance that one of the ellipses had changed its orientation by an angle drawn from a uniform distribution over all possible orientations. Following the second display, the observer pressed a key to indicate whether there was a change between the first and second displays. A response caused the next trial to begin. No trial-by-trial feedback was given. A practice block was identical to a testing block, except that all stimuli on a given trial had the same reliability, which was varied randomly across trials. Stimulus presentation time was initially 333 ms and decreased by 33 ms every 32 trials, allowing the observer to easy into the task. Feedback was given on each trial. The practice session consisted of 256 trials. A threshold block was identical to a practice block but used only the shortest stimulus presentation time (100 ms), and was 400 trials in length.

At the beginning of each session, subjects were informed in lay terms about the distributions from which the stimuli were drawn (e.g., “The change is equally likely to be of any magnitude.”). Each observer completed three sessions on separate days. The first session began with a practice block only for naïve subjects. All subjects then did one threshold block of 400 trials. We fitted a cumulative normal distribution to accuracy as a function of ellipse elongation and extrapolated the performance to the maximal elongation. If the resulting performance was equal to or greater than 75%, we found the elongation of a “short” ellipse from the 65% correct point of the fitted curve. If the resulting extrapolated performance was lower than 75%, the observer repeated the threshold block. If extrapolated performance on the repeated block was again lower than 75%, the observer was excluded from the study. Testing blocks had 400, 800, and 800 testing trials per session, respectively. There were two timed breaks spread evenly for the 400-trial session and four in the 800-trial ones. During each break, a screen showing the percentage correct in the block was displayed. Cumulative performance was shown at the end of each session.

### Encoding Model

For convenience, all orientations were remapped from [−π/2,π/2) to [−π,π). For a true stimulus orientation *θ*, we assumed the measurement *x* to follow a Von Mises distribution, 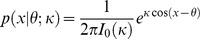, where *κ* is the concentration parameter. *κ* is determined by the amount of resource allocated to the stimulus, *J*. The relationship between *J* and *κ* is 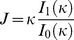, where *I*_1_ is the modified Bessel function of the first kind of order 1 (see Text S1). In the EP model, *J* is determined by ellipse elongation only. In the VP model, *J* is drawn from a gamma distribution with mean 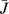 and scale parameter *τ*, where 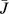 is determined by ellipse elongation (it is accordingly denoted 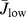 or 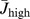).

### Model Predictions

We are interested in computing the probability predicted by a model of reporting “change” for a set of stimuli and corresponding reliabilities, given a set of parameter values. This probability is equal to the probability that *d*>1 for measurements (**x**,**y**) drawn using the generative model with the given parameters. This probability only depends on the magnitude of change, Δ, the number of high-reliability stimuli, *N*_H_, and whether a change, if any, occurred in a low-reliability or a high-reliability stimulus. We binned Δ every 3 degrees between 0 and 90 degrees, resulting in 31 values; *N*_H_ takes 5 possible values, resulting in 31⋅5⋅2 = 310 trial types. For each trial type, we approximated the distributions of **x** and **y** using a Monte Carlo simulation with 1,000 samples. For each sample, the model’s decision rule was applied, and the proportion of “change” responses among all samples was determined. This returned an estimate of the model’s probability of reporting “change” on a given trial, for the given parameter values. The entire procedure was repeated for all parameter combinations.

### Model Fitting

For a given model, we denote the vector of model parameters by **t**. The likelihood of **t** is the probability of the human subject’s empirical responses given **t**:
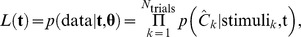



where *N*_trials_ is the total number of trials, 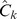 the subject’s response on the *k* ^th^ trial, and stimuli*_k_* is shorthand for the stimulus orientations and their reliabilities in both displays. The maximum-likelihood estimate of the parameters is the value of **t** that maximizes *L*(**t**).

### Bayesian Model Comparison

Each model *m* produces a prediction about the response on each trial, *p*(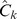
|stimuli*_k_*,**t**,*m*). Bayesian model comparison [34] consists of calculating for each model the probability of finding a subject’s actual responses under this distribution, averaged over free parameters:



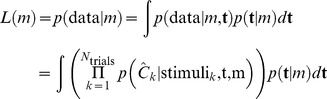



It is convenient to compute the logarithm of *L*(*m*) and write it as.
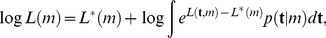
(4)


where 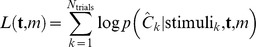 and 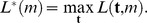 This form prevents numerical problems, since the exponential in the integrand of Eq. (4) is now of order 1 near the maximum-likelihood value of **t**. For the parameter prior, we assume a uniform distribution across some range, whose size we denote *R_j_* for the *j* ^th^ parameter. Ranges were as follows: [1,100] for *J*_low_, *J*_high_, *J*_assumed_, 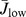, and 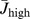; [1], [30] for *τ*; [−2.2, 51.8] for the Max model criterion *k*; [0.3, 0.7] for *p*_change_. Eq. (4) becomes 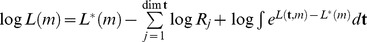, where dim **t** is the number of parameters. We approximated the integral through a Riemann sum. We tested the parameter fitting and model comparison code on fake data generated from each of the 14 models; parameters were estimated correctly and the model used to generate the data always won, showing that the models are distinguishable using this method.

## Supporting Information

Figure S1 **Encoding precision as a function of the concentration parameter of the Von Mises distribution.** The dashed line is the identity line.
(TIFF)

Table S1 **Parameter values for all models.** Mean and s.e.m. are over subjects.
(DOCX)

Text S1 **Supporting information.** Contains: Relationship between precision and concentration parameter; Equal-precision and variable-precision models; Optimally inferring change occurrence; The Max model
(DOCX)
