# Supplementary figures and images for: Probabilistic Computation in Human Perception under Variability in Encoding Precision
Source: PLoS One. 2012 Jun 29;7(6):e40216. doi: 10.1371/journal.pone.0040216 (PMC3387023; doi:10.1371/journal.pone.0040216)

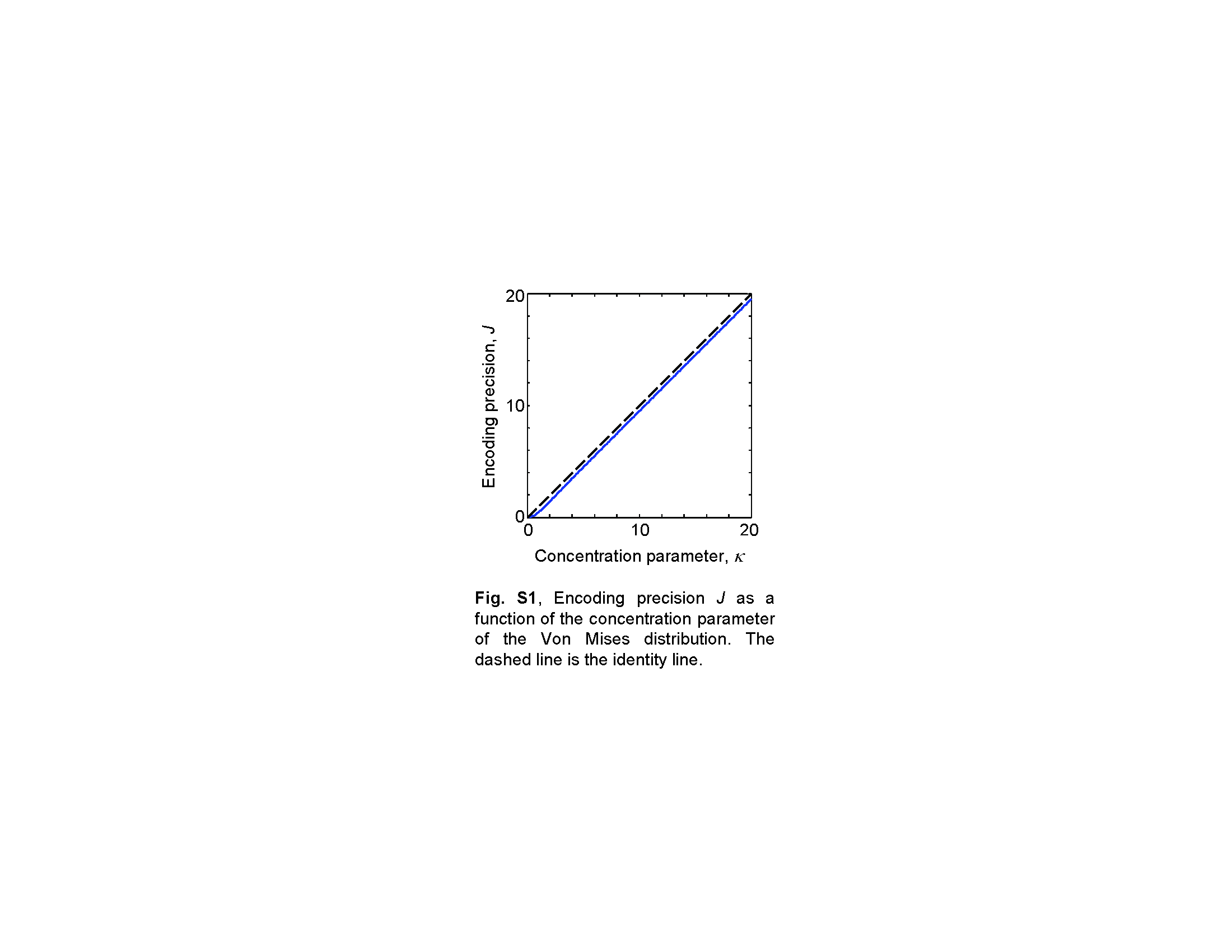

Supplement: Figure S1 — Encoding precision as a function of the concentration parameter of the Von Mises distribution. The dashed line is the identity line. (TIFF) [file pone.0040216.s001.tiff]
