# Supplementary material for: Probabilistic Computation in Human Perception under Variability in Encoding Precision
Source: PLoS One. 2012 Jun 29;7(6):e40216. doi: 10.1371/journal.pone.0040216 (PMC3387023; doi:10.1371/journal.pone.0040216)
Supplement: Table S1 — Parameter values for all models. Mean and s.e.m. are over subjects. (DOCX) [file pone.0040216.s002.docx]

**Table S1**: Parameter values for all models. Mean and s.e.m. are over subjects.

|  | **Variable-precision** | | **Fixed-precision** | |
| --- | --- | --- | --- | --- |
|  | **Optimal** | **Max** | **Optimal** | **Max** |
| **VP assumption** |  | |  | |
| *J*_low_ | 6.6 ± 1.4 | 7.5 ± 0.8 |  |  |
| *J*_high_ | 22 ± 3 | 21 ± 1 |  |  |
| *τ* | 21 ± 2 | 24 ± 3 |  |  |
| *k* | N/A | -0.44 ± 0.06 |  |  |
| *p*_change_ | 0.47 ± 0.02 | N/A |  |  |
| **FP assumption** |  | | | |
| *J*_low_ | 1.6 ± 0.2 | 2.8 ± 0.7 | 1.3 ± 0.1 | 1.5 ± 0.1 |
| *J*_high_ | 7.9 ± 1.6 | 9.8 ± 3.6 | 5.3 ± 1.2 | 5.4 ± 1.2 |
| *τ* | 1.7 ± 0.7 | 2.7 ± 1.2 | N/A | N/A |
| *k* | N/A | 0.94 ± 0.44 | N/A | -0.11 ± 0.07 |
| *p*_change_ | 0.36 ± 0.02 | N/A | 0.43 ± 0.01 | N/A |
| **AP assumption** |  |  |  |  |
| *J*_low_ | 2.5 ± 0.3 | 9.5 ± 2.0 | 1.8 ± 0.1 | 1.9 ± 0.2 |
| *J*_high_ | 5.0 ± 0.5 | 18 ± 5 | 3.5 ± 0.3 | 3.5 ± 0.3 |
| *τ* | 1.3 ± 0.2 | 16 ± 4 | N/A | N/A |
| *k* | N/A | 5.1 ± 1.2 | N/A | 0.01 ± 0.09 |
| *p*_change_ | 0.32 ± 0.01 | N/A | 0.39 ± 0.01 | N/A |
| **SP assumption** |  |  |  |  |
| *J*_low_ | 3.5 ± 0.3 | 3.3 ± 0.2 | 2.1 ± 0.2 | 2.0 ± 0.1 |
| *J*_high_ | 4.2 ± 0.4 | 4.3 ± 0.4 | 2.7 ± 0.4 | 2.7 ± 0.2 |
| *τ* | 1.5 ± 0.4 | 1.6 ± 0.2 | N/A | N/A |
| *J*_assumed_ | 1.5 ± 0.4 | N/A | 1.9 ± 0.2 | N/A |
| *k* | N/A | -0.86 ± 0.08 | N/A | -0.86 ± 0.08 |
| *p*_change_ | 0.50 ± 0.01 | N/A | 0.44 ± 0.03 | N/A |
