# Supplementary material for: Probabilistic Computation in Human Perception under Variability in Encoding Precision
Source: PLoS One. 2012 Jun 29;7(6):e40216. doi: 10.1371/journal.pone.0040216 (PMC3387023; doi:10.1371/journal.pone.0040216)
Supplement: Text S1 — Supporting information. Contains: Relationship between precision and concentration parameter; Equal-precision and variable-precision models; Optimally inferring change occurrence; The Max model (DOCX) [file pone.0040216.s003.docx]

**Probabilistic computation in human perception under variability in encoding precision**

**Supporting Information**

Shaiyan Keshvari, Ronald van den Berg, Wei Ji Ma

Baylor College of Medicine, Houston, TX, USA

# Relationship between precision and concentration parameter

For convenience, we remap all orientations from [−π/2, π/2) to [−π, π). We assume that the measurement *x* of a stimulus orientation *θ* follows a Von Mises distribution with concentration parameter *κ*:

.

We identify encoding precision with Fisher information, *J*(*θ*), which measures the performance of the best possible unbiased decoder. Substituting Eq. into the definition of Fisher information, we find

$J=\kappa\frac{I_{1}\left(\kappa\right)}{I_{0}\left(\kappa\right)}$.

*J* is a monotonically increasing function of *κ* and therefore invertible. It is shown in Fig. S1. The equivalent relationship for a Gaussian distribution would be *J*=1/*σ*^2^.

# Equal-precision and variable-precision models

In the equal-precision (EP) model, the encoding precision of an observation, *J*, is determined only by the elongation of the ellipse producing the observation. In the variable-precision (VP) model, encoding precision is a random variable that follows, independently for each item and each trial, a gamma distribution with mean $\bar{J}$and scale parameter *τ*, denoted $p\left(J|\bar{J};\tau\right)$. Variance is equal to $\bar{J}\tau$. The mean $\bar{J}$ differs between a long and a short ellipse. Thus, in this model, a measurement *x* is drawn from a doubly stochastic process: first precision is drawn from $p\left(J|\bar{J};\tau\right)$, then the measurement from the Von Mises distribution in Eq. with *κ* determined by *J* through Eq. .

# Optimally inferring change occurrence

The generative model (statistical structure) of the task is shown in Fig. 2c. We denote by *C* the binary variable of change occurrence (0: no change; 1: change), with a true prior *p*(*C*) =0.5. The orientations in the first display, **θ**=(*θ*_1_,…,*θ_N_*), are drawn independently from a uniform distribution over orientation space, and therefore we have

$p\left(\mathbf{\theta}\right)=\left(\frac{1}{2\pi}\right)^{N}$.

We denote by **Δ** the vector of change magnitudes at all locations. It is a vector of zeros except at the location where the change, of magnitude Δ, is located. Since all locations are equally likely to contain the change, we have

$p\left(\mathbf{\Delta}|C,\Delta\right)=\frac{1}{N}\sum_{i=1}^{N} \delta\left(\mathbf{\Delta}-C\Delta\mathbf{1}_{i}\right)$,

where **1***_i_* is the vector that has a 1 in the *i*^th^ entry and 0 everywhere else. The magnitude of change is drawn from a uniform distribution, *p*(Δ)=1/(2π). The orientations in the second display, **ϕ**=(*ϕ*_1_,…,*ϕ_N_*), are the sum of the initial orientations **θ** and the changes **Δ**:

$p\left(\mathbf{\varphi}|\mathbf{\Delta},\mathbf{\theta}\right)=\delta\left(\mathbf{\varphi}-\left(\mathbf{\Delta}+\mathbf{\theta}\right)\right)$.

Finally, the measurements in the first and second displays, **x**=(*x*_1_,…,*x_N_*) and **y**=(*y*_1_,…,*y_N_*), respectively, are drawn from independent Von Mises distributions with corresponding concentration parameters **κ***_x_*=(*κ _x_*_,1_,…, *κ_x,N_*) and **κ***_y_*=(*κ _y_*_,1_,…, *κ_y,N_*):

We have to consider the possibility that *κ_x_*,*_i_*≠*κ_y_*,*_i_*, because in the VP model, both sets of concentration parameters are independent random variables.

We are now ready to specify the observer’s inference process. Optimal inference amounts to “inverting” the generative model, i.e., to compute the probability of *C* given the noisy measurements of the orientations in the first and second displays on a given trial, **x** and **y**. The observer’s decision variable *d* is defined as the ratio between the probability that a change occurred and the probability that no change occurred, given **x** and **y**:

$$d=\frac{p\left(C=1|\mathbf{x},\mathbf{y}\right)}{p\left(C=0|\mathbf{x},\mathbf{y}\right)}=\frac{p\left(\mathbf{x},\mathbf{y}|C=1\right)}{p\left(\mathbf{x},\mathbf{y}|C=0\right)}\frac{p\left(C=1\right)}{p\left(C=0\right)},$$

where *p*(*C*) is the prior over *C*. The ideal observer will respond “change” (*Ĉ*=1) if *d*>1 and “no change” (*Ĉ*=0) when *d*<1. The joint distribution of **x**, **y**, and *C* is obtained by marginalizing the joint distribution of all variables over Δ, **Δ**, **θ**, and **ϕ**. It is most convenient to first marginalize over Δ, **Δ,** and substitute their distributions:

where in the last equality we have used the fact that given the vector **Δ**, the pairs of observations (*x_i_*,*y_i_*) are conditionally independent across locations. Substituting Eq. into Eq. and denoting the prior probability of *C*=1 by *p*_change_ gives

We now notice that the products in the numerator and denominator almost cancel, leaving only the *i*^th^ term:

.

The summand is exactly equal to the log likelihood ratio of a change occurring at the *i*^th^ location, so we can write:

$d=\frac{p_{\text{change}}}{1-p_{\text{change}}}\frac{1}{N}\sum_{i=1}^{N} d_{i}$,

where the local decision variable *d_i_* is defined as

.

We can evaluate *d_i_* by marginalizing over *θ_i_* and *ϕ_i_* and substituting the Von Mises noise distributions:

Substituting this result back into Eq. , we arrive at our final expression for the optimal decision variable:

$d=\frac{1}{N}\frac{p_{\text{change}}}{1-p_{\text{change}}}\sum_{i=1}^{N} \frac{I_{0}\left(\kappa_{x,i}\right)I_{0}\left(\kappa_{y,i}\right)}{I_{0}\left(\sqrt{\kappa_{x,i}^{2}+\kappa_{y,i}^{2}+2\kappa_{x,i}\kappa_{y,i}\cos\left(y_{i}-x_{i}\right)}\right)}$.

The observer reports “change” when *d* exceeds 1. As one would expect, the decision depends on the circular differences between the measurements in the first and second displays, *y_i_*−*x_i_*. The local concentration parameters in both displays, *κ_x,i_* and *κ_y,i_*, weight – albeit in a way that looks a bit less familiar than in cue combination with Gaussian distributions – the contribution of the evidence at each location to the decision.

The suboptimal assumptions about precision – average precision and single precision, as well as equal precision when precision is in reality variable – enter through modifications of Eq. . For the average-precision assumption, each *κ_x_*,*_i_* is replaced by an average over locations, $\bar{\kappa}_{x}$, and similarly each *κ_y_*,*_i_* by $\bar{\kappa}_{y}$. These numbers still vary from trial by trial and might be interepreted as a “gist” representation of precision. (We average concentration parameters *κ* rather than precision values *J* for convenience, but in view of the nearly linear relationship shown in Fig. S1, these are almost equivalent.) For the single-precision assumption, each *κ_x_*,*_i_* and *κ_y_*,*_i_* in the decision variable, Eq. , are replaced by a single assumed concentration parameter, *κ*_assumed_*.*

# The Max model

The decision rule in the Max model is max*_i_* *d_i_*>*k*, where *d_i_* is the local decision variable from Eq. and *k* is a criterion. When the local decision variable is modified using the assumption that the encoding precision of a stimulus has a single value throughout the experiment (which is a wrong assumption in our experiment already because stimulus reliability can be low or high), then the Max decision rule simplifies. This is because max*_i_* *d_i_* can be rewritten as

$$\max_{i} \frac{I_{0}\left(\kappa_{\text{assumed}}\right)^{2}}{I_{0}\left(\kappa_{\text{assumed}}\sqrt{2+2\cos\left(y_{i}-x_{i}\right)}\right)}=\frac{I_{0}\left(\kappa_{\text{assumed}}\right)^{2}}{I_{0}\left(\kappa_{\text{assumed}}\sqrt{2+2\min_{i} \cos\left(y_{i}-x_{i}\right)}\right)},$$

where we have used the facts that *κ*_assumed_ is a constant and *I*_0_ is a monotonically increasing function. The resulting rule can be rewritten as a condition on min*_i_* cos(*y_i_*−*x_i_*):

$$\min_{i} \cos\left(y_{i}-x_{i}\right)<\tilde{k}$$

where $\tilde{k}=\frac{1}{2}\left(\frac{1}{\kappa_{\text{assumed}}}I_{0}^{-1}\left(\frac{I_{0}\left(\kappa_{\text{assumed}}\right)^{2}}{k}\right)\right)^{2}-1$ is a different criterion. This proves the equivalences in Table 1. Note that *κ*_assumed_ and *k* both only appear in the model through this decision rule, and can therefore not be fitted independently : they are effectively a single parameter. Eq. is equivalent to comparing the largest circular distance between two observations at the same location to a criterion:

,

where |⋅| denotes the unsigned distance along the circle. In earlier work, we called this suboptimal model the *maximum-absolute-differences model*^1^. In that paper, stimulus reliability was not deliberately varied and this model would be closer to optimal than it is in the present experiment.

**References**

1. Wilken, P. & Ma, W.J. A detection theory account of change detection. *J Vision* **4**, 1120-1135 (2004).
